# The Role of Tumor Tissue Architecture in Treatment Penetration and Efficacy: An Integrative Study

**DOI:** 10.3389/fonc.2013.00111

**Published:** 2013-05-10

**Authors:** Katarzyna A. Rejniak, Veronica Estrella, Tingan Chen, Allison S. Cohen, Mark C. Lloyd, David L. Morse

**Affiliations:** ^1^Integrated Mathematical Oncology, H. Lee Moffitt Cancer Center and Research Institute Tampa, FL, USA; ^2^Department of Oncologic Sciences, College of Medicine, University of South Florida Tampa, FL, USA; ^3^Department of Cancer Imaging and Metabolism, H. Lee Moffitt Cancer Center and Research Institute Tampa, FL, USA; ^4^Analytic Microscopy Core Facility, H. Lee Moffitt Cancer Center and Research Institute Tampa, FL, USA; ^5^Department of Biological Sciences, University of Illinois at Chicago Chicago, IL, USA; ^6^Department of Physics, College of Arts and Sciences, University of South Florida Tampa, FL, USA

**Keywords:** interstitial transport, tissue penetration, drug/biomarker efficacy, regularized Stokeslets Method, intravital fluorescence microscopy, mouse xenograft tumor model, tumor targeted agent, fluorescence molecular imaging

## Abstract

Despite the great progress that has been made in understanding cancer biology and the potential molecular targets for its treatment, the majority of drugs fail in the clinical trials. This may be attributed (at least in part) to the complexity of interstitial drug transport in the patient’s body, which is hard to test experimentally. Similarly, recent advances in molecular imaging have led to the development of targeted biomarkers that can predict pharmacological responses to therapeutic interventions. However, both the drug and biomarker molecules need to access the tumor tissue and be taken up into individual cells in concentrations sufficient to exert the desired effect. To investigate the process of drug penetration at the mesoscopic level we developed a computational model of interstitial transport that incorporates the biophysical properties of the tumor tissue, including its architecture and interstitial fluid flow, as well as the properties of the agents. This model is based on the method of regularized Stokeslets to describe the fluid flow coupled with discrete diffusion-advection-reaction equations to model the dynamics of the drugs. Our results show that the tissue cellular porosity and density influence the depth of penetration in a non-linear way, with sparsely packed tissues being traveled through more slowly than the denser tissues. We demonstrate that irregularities in the cell spatial configurations result in the formation of interstitial corridors that are followed by agents leading to the emergence of tissue zones with less exposure to the drugs. We describe how the model can be integrated with *in vivo* experiments to test the extravasation and penetration of the targeted biomarkers through the tumor tissue. A better understanding of tissue- or compound-specific factors that limit the penetration through the tumors is important for non-invasive diagnoses, chemotherapy, the monitoring of treatment responses, and the detection of tumor recurrence.

## Introduction

Systemic chemotherapy is one of the main anticancer treatments used for most kinds of tumors that are clinically diagnosed. However, with a few exceptions, such as the treatment of chronic myeloid leukemia with *imatinib*, the drugs that have shown high promise for a cure in laboratory tests did not prove to be as successful in the clinical setting. In fact, only about 10% of the drugs that enter clinical trials are approved by the FDA (Petsco, 2010), and the majority of potentially therapeutic compounds fail in Phase II of the clinical trials. This means that the drugs are not effective in treating the disease, even though they were potent in cell-culture assays and animal model systems (Petsco, 2010). One of the reasons for the Phase II drug failures may be attributed to the fact that experimental models do not recreate the process of interstitial drug transport in the tissues in the same way that it occurs in the patient’s body. It is beyond question that even the most effective anticancer drug will not show high efficacy if it cannot reach all of the tumor cells in concentrations sufficient to exert a therapeutic effect. Moreover, it has been suggested (Minchinton and Tannock, 2006) that the poor penetration of the tumor tissue by drug particles may leave untreated certain cell populations capable of initiating tumor recurrence and/or resistance.

Recent advances in molecular imaging allow for the development of targeted imaging agents that are specific in binding to intracellular or extracellular targets (biomarkers). They can predict the pharmacological responses to therapeutic interventions and are being used during the diagnoses to determine the state of the disease and to plan (personalized) treatment. Imaging biomarkers that allow for the prediction and monitoring of patient responses to a given therapy are becoming an essential component of drug development. Moreover, they can reduce the number of patients needed to test novel targeted therapeutic agents by identifying non-responders early-on. However, the transport of such biomarkers through the tumor tissue faces similar issues. In order to be a useful predictor, targeted imaging agents need to access the tumor tissue space and then be retained by individual cells through binding and uptake (Morse and Gillies, 2010).

**Figure 1:**
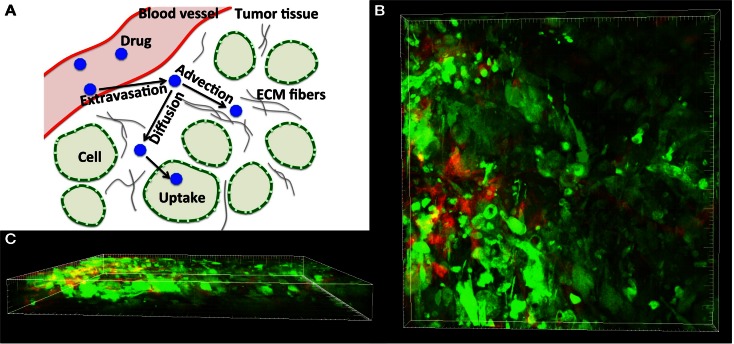
**Tumor tissue structure as a barrier in drug and imaging agent efficacy**. **(A)** Schematics of the complex dynamics of drug/imaging molecule movement through the tissue, including extravasation, diffusive and advective transport, and internalization. **(B)** Three-dimensional multiphoton emission (MPE) microscopy image reconstruction (400× magnification) of the human MDAMB231/GFP xenograft tumor from the dorsal-skin fold window chamber. **(C)** Horizontal view of **(B)** showing the depth in the tissue structure. Staining: green-tumor cells, red-vasculature.

Drug penetration refers to the movement of drug molecules from the bloodstream into the various tissues of the body (Minchinton and Tannock, 2006). After a drug is absorbed into the bloodstream, it rapidly circulates through the body; however, both the spatial and temporal distributions of the drug molecules may be different in different tissues types and the extent of the drug/biomarker particle penetration into the tissue depends on both the biochemical properties of the particles and biophysical properties of the tissues. For example, drugs that dissolve in water (water-soluble drugs) tend to stay in the bloodstream and in the fluid that surrounds the cells (interstitial fluid). Particles have different sizes and molecular weights and thus their penetration into the tumor tissue may depend on whether their transport through the interstitial space is dominated by their random motion (diffusion) or motion due to the fluid flow (advection) (Jain, 1987; Gade et al., 2009; Schmidt and Wittrup, 2009). The interstitial transport of drug molecules may also be affected by the tumor cellular structure (Grantab et al., 2006) and extracellular matrix (ECM) assembly (Netti et al., 2000). We are particularly interested in the cellular architecture of tumor tissue, which may be highly unorganized, irregular, and heterogeneous (Figure 1), and in the role that the size of the extracellular space between the cells plays in interstitial transport by both diffusion and interstitial fluid advection. We will investigate the complex interplay between these processes of extracellular transport and drug penetration.

Most *in silico* models applied to drug development use bio-statistics and bio-informatics methods to screen large numbers of therapeutic compounds. The pharmacokinetic (PK) properties of the drugs are then determined by fitting the actual data to a theoretical compartmental model, followed by rigorous “goodness-of-fit” test statistics (Michelson et al., 2006). Although numerous computational methods have been developed for the *in silico* testing of various properties of drug particles (known under the acronym ADME-T: absorption, distribution, metabolism, excretion, and toxicity (Beresford et al., 2002; Boobis et al., 2002; Ekins and Rose, 2002; Kerns and Di, 2008; Huynh et al., 2009) they do not consider the spatial aspects of drug PKs and treat all organs as well-mixed compartments neglecting their natural heterogeneities. Thus, the poor penetration of the tumor tissue as a limiting factor for drug efficacy is not currently included in a typical ADME-T protocol.

Mathematical PK models that include tissue transport phenomena are usually defined as continuous mixture models with the tumor tissue being represented by a homogeneous material (Baxter and Jain, 1989; Jackson and Byrne, 2000; Zhao et al., 2007; Sinek et al., 2009; Shipley and Chapman, 2010). These models showed importance of the kinetics of the drug supply from the blood system, as well as its diffusive and advective transport, on the concentration profiles of biochemical compounds, and the significant impact of nutrient distribution on the drug’s therapeutic efficacy. However, they have not addressed the heterogeneity of the tumor cells, or the transport of individual drug/biomarker particles. These aspects will be incorporated in the mechanistic model described in this paper that is based on the fluid-structure interaction method of the regularized Stokeslets (Cortez, 2001). We take into account, explicitly, the cellular structure of the tumor tissue, and investigate how the tumor tissue composition influences the interstitial transport of chemical compounds. In particular, we analyze the relationship between the cellular porosity and/or cellular density of the tissue at the depth at which the drug/biomarker particles penetrate it. Our computational results are also compared to the experimental data showing the differences in the penetration and uptake of targeted imaging agents in tumors that express the cell-surface receptor of interest (positive tumors) or not (negative tumors). This study will offer an insight into the potential mechanisms preventing the adequate delivery of anticancer drugs.

## Materials and Methods

### The mathematical model

**Figure 2:**
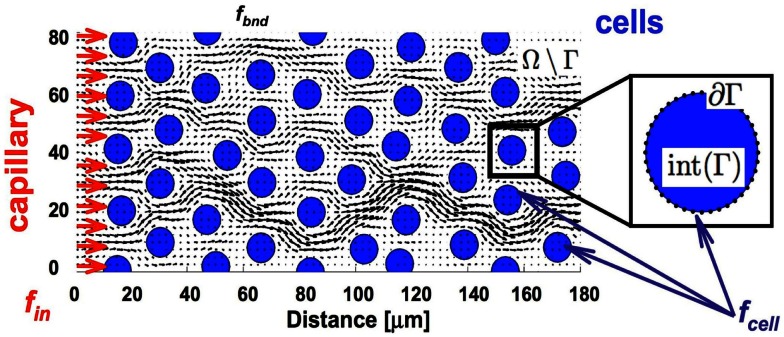
**Tissue scheme in the model**. Schematic representation of the computational domain containing several tumor cells (blue circles) surrounded by the interstitial fluid (black arrows) that is supplied from the capillary located at the left border of the domain (red arrows). Adequate forces (*f*_in_ and *f*_bnd_) are chosen to create the physiological fluid flow and to keep the boundaries of all tumor cells immobile (*f*_cell_).

We consider here a small (a few hundred of microns in length) two-dimensional patch of the tumor tissue (Ω) with explicitly defined tissue morphology composed of individual tumor cells (Γ = ∑ Γ*_i_* where *i* = 1, …, *N_b_*, and *N_b_* is the number of cells) embedded in the ECM and surrounded by interstitial space filled with fluid (Ω\Γ, Figure 2). The reported experimental measurements of the interstitial fluid velocities are in the order of 0.1–2 μm/s (Chary and Jain, 1989; Swartz and Fleury, 2007), thus the simulated time needed for drug particles to transverse the modeled tissue is in the order of a few minutes. Therefore, we treat all cells as stationary, i.e., we assume that during the simulation time the cells are immobile and will not grow, divide, or die (thus the cell shapes and positions are fixed). Moreover, since the characteristic cell-tissue length scale is in the order of 10–100 μm, the corresponding Reynolds number is small (*Re* = ρ*LV*/μ = 10^−7^ to 10^−5^, where ρ is the fluid density, μ is the fluid viscosity, and *L* and *V* are the characteristic length and velocity scales, respectively). Hence, the fluid flow can be approximated by the Stokes equations:

(1) $$\begin{array}{ll} \mu \;\Delta u\left(x\right) & =\nabla p\left(x\right)-f\left(x\right), \end{array}$$

(2) $$\nabla \cdot u\left(x\right)=0,$$

where *p* is the pressure, *u* is the fluid velocity, and *f* = *f*_in _+ *f*_bnd _+ *f*_cell_, is the force applied to the domain edges ∂Ω(*f*_in_, *f*_bnd_), and cell boundaries ∂Γ(*f*_cell_) to create the physiologically relevant interstitial fluid flow and to keep the cells immobile. These equations are solved using the classical fluid-structure interaction method of the regularized Stokeslets (Cortez, 2001). In this method, each force *f* concentrated at a single point *x*_0_ is smoothed over a small ball of radius ε using a cut-off function Φ_ε_, that is *f*(*x*) = *f*_0_Φ_ε_(*x *− *x*_0_). The cut-off function needs to be radially symmetric, vary smoothly from its maximal value at the center to zero at the surface, and satisfy the condition: $\int \phi_{\varepsilon }\left(x\right)dx=0.$ We follow (Tlupova and Cortez, 2009) and use the function $\phi_{\varepsilon }=\frac{2\varepsilon^{4}}{\pi {\left({∥x∥}^{2}+\varepsilon^{2}\right)}^{3}}$for which the regularized Stokes equations have the exact solution. Other examples of suitable cut-off functions can be found in Cortez (2001) and Tlupova and Cortez (2009) together with their detailed derivation. Since the Stokes equations are linear, one may represent the fluid flow as a direct summation of the contributions from finitely many discrete forces *f_k_*, which gives the following expression for the fluid velocity *u* that we will use in all our simulations (N is the number of forces):

(3) $$\begin{array}{llll} u\left(x\right)= & \overset{N}{\underset{k=1}{\sum }}\left\{-\frac{1}{4\pi \mu }f_{k}\left(\text{ln}\left(r^{2}+\varepsilon^{2}\right)-\frac{2\varepsilon^{2}}{r^{2}+\varepsilon^{2}}\right)\right. & & \\ & +\left.\frac{1}{4\pi \mu }\frac{1}{r^{2}+\varepsilon^{2}}\left[f_{k}\cdot \left(x-x_{k}\right)\right]\left(x-x_{k}\right)\right\}, \end{array}$$

where $r_{k}=∥x-x_{k}∥$. The regularization parameter ε has been chosen to be equal to the cell boundary point separation that is optimal for reducing the regularization error (Cortez et al., 2005; Tlupova and Cortez, 2009). In this model we assume that the fluid is supplied from the capillary located at the left boundary of the domain, and penetrates the interstitial space around the tumor cells (Ω\Γ) as shown in Figure 2.

The individual molecules (of the drug or imaging agents under consideration) are modeled as a collection of discrete particles *y_p_* that enter the tissue via the transmural influx from a capillary (together with the interstitial fluid), and advance through the tissue by a combination of advective transport, with the fluid flow calculated using the regularized Stokeslets method, and diffusion modeled as Brownian motion with a diffusion coefficient *D* and a randomly chosen direction of movement ϖ. Particle movement (without cellular uptake) is confined to the extracellular space (Ω\Γ) only. The advective transport satisfies this condition since the fluid flow is zero at all cell boundaries. For the diffusive movement we ensure that the particles do not cross the cell membrane incidentally by maintaining their old positions whenever the randomly chosen direction of movement would push the particles into the intracellular space (Eq. 4, parameter α). The process of particle internalization by the cells is modeled separately by trapping the particle by the near-by cell boundary receptor if the particle comes close to the cell boundary (*X_l_* within a small distance δ, Eq. 4). This is a very simplified way to model drug uptake, and results in 100% binding rate. However, molecular binding is usually not so efficient, and depends not only on the distance between the receptor and the ligand but also on the chemical or electrostatic forces between the two. The numerical procedure for updating the positions of the drug/imaging agent particles is given in Eq. 4,

(4) $$\begin{array}{llll} y_{p}^{n+1} & =\left\{\begin{array}{cc} X_{l},\; & \;\text{if }∥y_{p}^{n}-X_{l}∥\leq \delta \&X_{l}\in \partial \Gamma ,\\ & \;\delta <<\varepsilon \\ y_{p}^{n}+\alpha \left(u\left(y_{p}^{n}\right)\Delta t+\sqrt{2D\Delta t}\;\varpi^{n}\right),\; & \text{otherwise}\\ \; \end{array}\right., & & \\ \text{and }\alpha & =\left\{\begin{array}{cc} 1,\; & y_{p}^{n},y_{p}^{n+1}\in \Omega \backslash \Gamma ,\\ 0,\; & y_{p}^{n}\in \Omega \backslash \Gamma ,y_{p}^{n+1}\in \Gamma ,\\ 0,\; & y_{p}^{n}\in \Gamma .\\ \; \end{array}\right. \end{array}$$

and the physical and computational parameters of the model are listed in Table 1.

**Table 1:** **Model physical and computational parameters**.

Parameter	Symbol	Value
Domain	Ω	210 × 80 μm
Tissue cellular porosity	ψ	40–90%
Tissue cellular density	ξ	2–6 Cells per column
Interstitial fluid input flow	*u* ^in^	(1, 0) μm/s
Fluid viscosity	μ	2.5 × 10^3^ μg/(mm.s)
Regularization parameter	ε	0.5 μm
Discretization parameter		0.5 μm
Time step	Δ*t*	0.1 s
Diffusion coefficient	*D*	2.5 × 10^−8^ to 10^−3^ mm^2^/s
Direction and distance of motion	ϖ	0–360° and [0, √(2*D*Δ*t*)]
Uptake rate		100%
Binding distance	δ	0.5ε

The velocity in Eq. 3 can be used in two ways. First, for the given forces one can directly compute the induced velocity at any point in the domain. Second, since this equation is linear, one can use an iterative method, such as the generalized minimal residual method (*gmres*), to determine the forces that will result in the desired velocities at certain points in the domain (Cortez, 2001). Thus, our final computational algorithm can be summarized as follows: (1) use Eq. 3 to compute the forces (i) at the capillary which will result in the desired fluid and drug supply; (ii) on all cell boundaries to keep the cells immobile, and (iii) on the upper and lower edges of the computational domain to impose a zero flow there; (2) use Eq. 3 and the forces determined in (1) to compute the fluid velocities at all points representing the drug/imaging agent molecules; (3) use Eq. 4 to compute the new locations of all of the particles due to their advective transport at the local fluid velocity determined in (2), and their diffusive motion within the extracellular space. (4) Determine the cellular uptake of the particles, if the particles move close to the cell membrane receptors and the cells are capable of binding the particles. Repeat iteratively steps (2–4) to advance the particles through the tumor tissue.

### Intravital imaging of Dmt-Tic-Cy5 using the dorsal window chamber tumor xenograft model

A dorsal window chamber (DWC) xenograft tumor model was used to study the PKs of the tumor cell binding and uptake of the δ-opioid receptor (δOR) targeted fluorescent agent Dmt-Tic-Cy5 (Josan et al., 2009). HCT116/δOR colon cancer cells engineered to express the δOR on the cell-surface, or δOR negative HCT116 parental cells were mixed with rat GFP expressing microvessels and aseptically inoculated within the exposed epidermis of the dorsal chamber. Following tumor cell implantation, a glass cover was placed in the chamber to cover the xenograft tumor. Ten days after cell implantation, mice were intravenously injected with 100 μl of 5% 10,000 MW Cascade Blue Dextran (Invitrogen, CA, USA) in sterile H_2_O to verify microvessel patency. Then, 45 nmol/kg of the δOR specific Dmt-Tic-Cy5 probe was injected into the tail vein. Confocal fluorescence microscopy images were continuously acquired for a period of time, during and after the injection of the probe, using an Olympus FV1000 (MPE) Multiphoton Laser Scanning Microscope (Lisa Muma Weitz Advanced Microscopy and Cell Imaging facility at USF) with 250× magnification and an acquisition rate of 3570 pixels/min. The presence of Dmt-Tic-Cy5 was measured by excitation with a 635 nm wavelength laser and the emitted light was detected using a 655–755 nm emission filter. All procedures were carried out in compliance with the Guide for the Care and Use of Laboratory Animal Resources (1996), National Research Council, and were approved by the Institutional Animal Care and Use Committee (IACUC) at the University of South Florida.

### Histological imaging of human ovarian tumor

A sample of invasive ovarian tumor has been selected retrospectively from the Moffitt Cancer Registry database. A section of the formalin fixed and paraffin embedded (FFPE) tissue (4 μm thick) has been stained with a hematoxylin and eosin stain (H&E). The whole slide was scanned using the Aperio™ (Vista, CA, USA) ScanScope XT with a 20×/0.8 NA objective lens at a rate of 3 min per slide via Basler tri-linear-array. All procedures were carried out in compliance with HIPAA regulations with patient consent and were approved by the Institutional Review Committee (IRB # Pro00003491) at the University of South Florida and the Moffitt Cancer Center Scientific Review Committee (SRC # 16511). The original H&E-stained histological image has been digitized using the ImageJ software (NIH, USA) and in house Matlab routines. The digitized version has been used for computational simulations using the model described in Section “The Mathematical Model.”

## Results

Experimental evidence (Gullino et al., 1965; Nugent and Jain, 1984; Jain, 1987; Netti et al., 2000; Levitt, 2003; Grantab et al., 2006) has shown that tissue histology, cell packing density, and the extent of the ECM, and the amount of interstitial water can vary significantly between cancers of various origins (breast, brain, ovary, and lung). For example, in tumors grown in rats the interstitial intertumoral space can vary from around 35% in certain carcinosarcomas and carcinomas, to around 65% in fibrocarcinomas and sarcomas (Jain, 1987). Our goal is to investigate how the structure and cellular composition of tumor tissues influences the interstitial transport of chemical compounds, such as drug or biomarker molecules. We will use a suit of idealized computational tissues with various morphological parameters (cellular size, tissue porosity) and different properties of drug particles (diffusion coefficient, cellular absorption) to run computational simulations and compare the depth and timing of the molecule distributions within the tissue. This systematic exploration allows us to determine the relative importance of the physical parameters of both the tissue and the drug required for effective interstitial transport. Finally, we will compare our simulation outcomes with experimental results from tumor cells grown in the DWC and treated with targeted imaging agents to determine their spatial and temporal penetration dynamics.

### Permeation in idealized tissues of regular architecture

**Figure 3:**
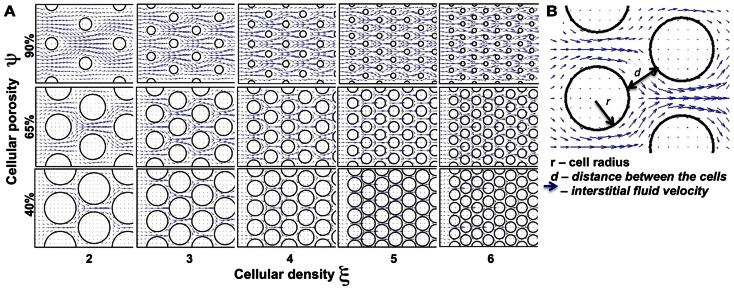
**Differences in the tissue’s cellular structure**. **(A)** Collection of tissue samples with regularly distributed cells with various values of cellular porosity ψ (defined as a percentage of void spaces in the tissue; here ψ = 40, 65, 90%) and cellular density ξ (determined by the number of cells in each column; here ξ = 2, 3, …, 6). Tumor cells are represented by circles and the interstitial fluid velocity field initiated at the left edge of the domain is shown as blue arrows pointing in the direction of fluid flow. **(B)** The interstitial space is determined by the size (*r*) of the cells and distance (*d*) between the neighboring cells.

We began our study by examining idealized computational tissues composed of identical regularly distributed circular cells. This allowed us to analyze the properties of the mechanistic model when tissue heterogeneity is ignored. Furthermore, we compared these results with cases where the regular tissue structure is perturbed. Since the interstitial transport takes place in the void space separating individual cells, the depth of tissue penetration depends on the relative volume of all voids, which we quantify as tissue cellular porosity, ψ. However, the particular pattern of the interstitial fluid flow for the fixed porosity value relies on the actual space between individual cells, and thus on the cell size and number (density) within the tissue. In the case of idealized tissues with regularly spaced circular cells we defined the tissue cellular density as the number of cells in each column, ξ. The subject of our investigation is the permeation time and penetration depth of the drug/biomarker particles transported by the interstitial fluid flow through a tissue with given morphological parameters (ψ, ξ). Figure 3 shows a collection of tissue samples with regularly distributed cells for the three porosity values of ψ = 40, 65, and 90%, and the cellular densities of ξ = 2, …, 6 (the pattern of the interstitial fluid flow in each case is shown in blue).

We examined a total of 30 cases of regular cellular patterns in which the tissue’s cellular porosity varied between 40 and 90%, and the tissue’s cellular density varied between two and six cells. In each *in silico* tissue, all cells had identical radii determined in such a way to reach the desired values of both the cellular porosity of the tissue and its cellular density. Note that in each row in Figure 3 the value of the tissue cellular porosity is fixed, and that for an increasing number of cells that occupy each column (cases ordered from left to right), the overall cellular density increases. However, it is not immediately clear how the tissue permeability, i.e., the extent of the interstitial fluid penetration of the tissue, is related to the tissue cellular structure. In fact, we will show that the less dense tissues may have lower permeation properties.

**Figure 4:**
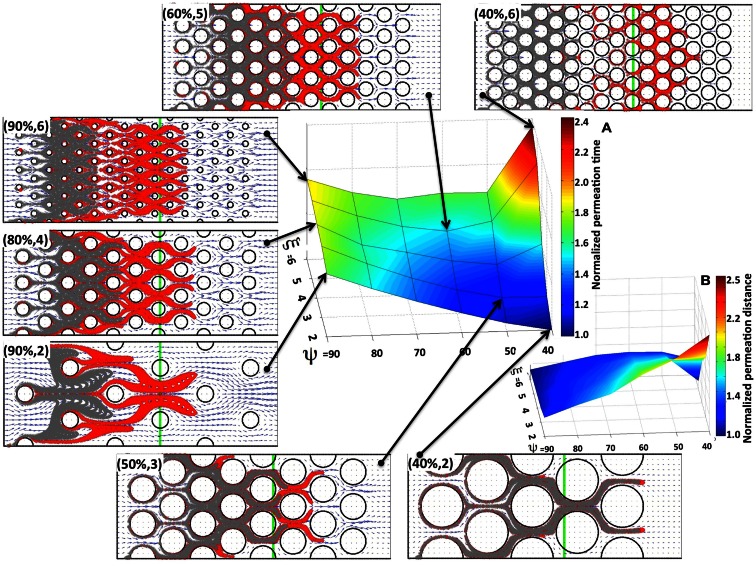
**Drug particle permeation times and depths for advective flow in regularly packed tumor tissues**. Surfaces showing **(A)** the relative permeation time of drug particles (the time required for a quarter of the supplied drug particles to reach 2/3 of the computational domain length), and **(B)** the normalized depth of tissue penetration at a fixed time for tissues of varying cellular porosity and cellular density (ψ, ξ). Seven specific tissue configurations of indicated cellular porosity and cellular density are shown in each case. The locations of drug particles at a fixed time from **(B)** are shown in gray, and their final positions at the permeation times from **(A)** are shown in red. The green vertical lines indicate the fixed distance at which the permeation time is recorded.

We first consider the advective transport only. In each case under consideration we computed the interstitial fluid field (Figure 3) that was a result of the steady fluid influx of the velocity *u*^in^ = (1, 0) μm/s (Swartz and Fleury, 2007) along a capillary located at the left edge of the domain. Subsequently, we introduced identical numbers of drug particles (*N_p_* = 4800) from uniformly spaced capillary fenestration, and traced the drug particles trajectories within the interstitial fluid flow during their transport. It is not known *a priori* whether all of the particles will be able to transverse the whole tissue patch, as there is the possibility that they may be carried with the flow to some tissue spots where the interstitial fluid velocity is very low or even zero (for example particles located near the cell boundaries). Therefore, to be able to compare the results across different tissue geometries we recorded the time (that we call the *permeation time*) when a certain fraction of the fastest drug particles (a quarter of all particles introduced to the system) reaches a prescribed distance from the capillary (we chose the distance of 120 μm, which requires the particles to travel 2/3 of the whole computational domain). The choice of both values, the distance at which the permeation time is measured and the fraction of particles to take into consideration are somewhat arbitrary, but our main goal is to compare the results between tissues of different properties using unified criteria. We do not expect the overall conclusions from our model simulations to be significantly different if we choose a different fraction of particles or a different distance from the capillary. The permeation time normalized by the minimal value across all 30 tissue samples is presented in Figure 4A as a surface plot. Here, the slowest permeation time for the tissue of (ψ, ξ) = (40%, 6) is more than twofold longer than the fastest permeation time for the tissue (ψ, ξ) = (40%, 2). Figure 4B shows a plot of the maximal distances reached by the drug particles at the fixed time equal to the minimal permeation time from Figure 4A. Seven particular tissue samples are shown in more detail (red points show final locations of drug particles at the permeation times from Figure 4A, gray points show locations of drug particles at the fixed time from Figure 4B). For the minimal permeation time (lower right inset) both the red and gray particles overlap.

As expected, for a given tissue cellular porosity the permeation time increases with increasing tissue cellular density as a result of the diminished space between neighboring cells (Figure 4A). However, for the cases when the cell column occupancy is small (ξ = 2, 3, 4) the tissues characterized by a lower cellular porosity, and thus with a smaller separation between neighboring cells, are traversed faster by the majority of the drug particles, and thus the corresponding permeation times are lower. The global minimum in permeation times occurs for the porosity ψ = 40% and cellular density ξ = 2. For higher cellular densities (i.e., larger numbers of cells occupying each column), the local minima in the permeation times occur at the middle-rank of a given tissue porosity, that is at ψ = 60%. This is also confirmed in Figure 4B, where the larger traveled distances are observed in the cases of either low cellular porosity or low cellular density (right lower corner in Figure 4B). These results were obtained under the assumption that the interstitial fluid influx *u*^in^ from the capillary is identical in all 30 *in silico* tissues considered here. As a consequence, the fluid velocity in denser tissues is higher, since the same amount of fluid is moving through a narrower space, and the drug molecule permeation time is faster.

### Permeation in idealized tissues of irregular architecture

**Figure 5:**
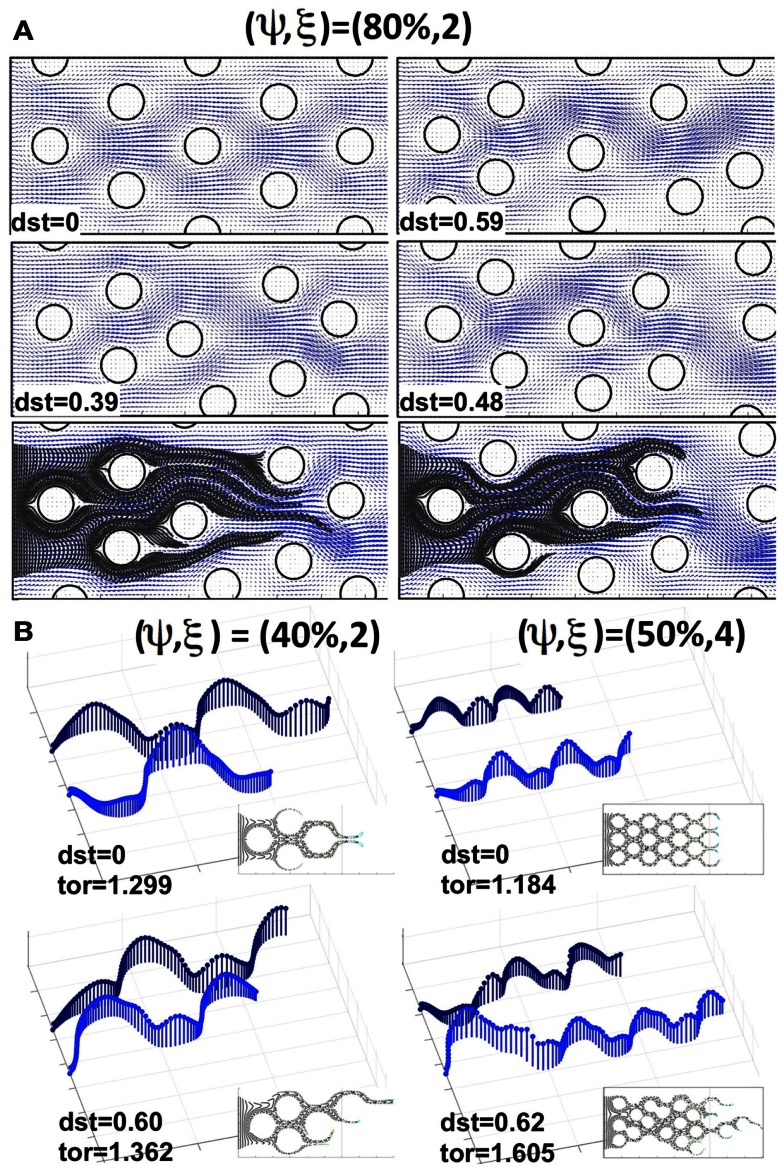
**Comparison of drug particle traces and tortuosity in regular and irregularly perturbed tissue geometries**. **(A)** A regularly packed tissue (upper left corner) and three cases of irregular geometries (upper right corner and middle row), all with a cellular porosity of ψ = 80% and cellular density of ξ = 2 cells, together with the resulting interstitial fluid velocity fields and indicated distortion (dst). Bottom row: traces of drug particles along the “corridors” with a higher interstitial fluid flow for the two irregular geometries shown in the middle row. **(B)** The effective paths of two representative drug particles (the same in each tissue) for a pair of regularly and irregularly packed tissues: (ψ, ξ) = (40%, 2) and (ψ, ξ) = (50%, 4). The length of each vector (blue) indicates the speed of a particle at the given position. Insets show traces of several other drug particles for the given tissue topology. The maximal tortuosity values (tor) for each tissue are provided.

Now we consider cases in which the cells are non-uniformly distributed within the tissue. We examined tissue geometries obtained by shifting the locations of the tumor cells with respect to the regularly ordered tissues. Figure 5A shows a regularly (upper left corner) and three irregularly packed tissues (upper right corner and both pictures in the middle row), all with cellular porosities of ψ = 80% and cellular densities of ξ = 2 cells per column (the irregular tissue geometries were obtained from the regular ones by randomly shifting the cell centers around their initial positions without cell overlap). These tissue irregularities result in the asymmetrical flow of the interstitial fluid (fluid velocity fields on the same grid are shown in blue in Figure 5A), and in the formation of interstitial “corridors” characterized by higher fluid flow. We quantify this distortion (on the scale of the whole tissue patch) by comparing the differences in the whole velocity fields between the irregularly and regularly packed tissues using the *L*_1_-norm, *dst* = ||*u*_reg_ − *u*_irreg_||_1_/(*N_x_* × *N_y_*), where *N_x_* × *N_y_* is the number of grid points upon which the fluid velocity field is evaluated (the distortion values for each tissue are shown in Figure 5). These emergent fluid corridors are followed by drug particles during their advective transport through the tumor interstitium (see Figure 5A bottom row).

The actual microscopic paths followed by the fluid flow carrying the drug particles can be geometrically complex. Thus, we use a quantitative metric of the drug particle path tortuosity in order to illustrate the differences between the individual routes of drug transport within the tissue. The path tortuosity τ is defined here as a ratio of the effective path length (*L_e_*) to the shortest straight-line distance (*L*) between the initial and final positions of the moving particle, that is τ = *L_e_*/*L*. Note that the tortuosity of a straight line is 1, of a circular path is infinity, and that it has been estimated in Ramanujan et al. (2002) that the tortuosity of a well-packed system of cells is τ = √2. Therefore, the determination of the mean and maximal tortuosity of individual drug particle paths can provide a better understanding of the void space complexity inside the tumor tissue and the patterns of the interstitial fluid flow. The average tortuosities under the advective flow taken over all 30 different regular and 150 irregular tissues considered in our simulations are similar (τ = 1.12 ± 0.05 and τ = 1.20 ± 0.07, respectively). However, for every irregularly packed tissue we have observed some drug particle paths of tortuosity above √2, and the average maximal tortuosity in these tissues is τ = 1.64 ± 0.3. Thus, the irregular tissue topology implies that some drug particles traverse across the tissues in a very complex way. Figure 5B shows the representative traces of the same drug particles for two pairs of tissues, one regularly and one irregularly packed. The case of minimal overall permeation (ψ, ξ) = (40%, 2) is shown in the left column, whereas the case of the highest average tissue tortuosity (ψ, ξ) = (50%, 4) is presented in the right column. The speed of each traced particle at every visited position inside the tissue is indicated by the length of the vertical vector. These values depend strongly on the cellular structure of the tissue, and oscillate around the cell perimeters. However, in the case of regular tissues these oscillations are periodic. Thus, when the particles are supplied through uniformly spaced capillary fenestration, they can cover the whole tissue width evenly. In contrast, in the irregular tissues the drug particles may travel across the width of the tissue by utilizing the fluid flow corridors, and even if the drug particles initial locations along the capillary were distinct, they may end up following the same path. Moreover, the speed of the drug particles in these cases is very non-homogeneous (as seen in Figure 5B, left lower image), and the tortuosities of many of the particles’ paths are above √2, in contrast to the regular cases. This causes a non-uniform exposure of the drug to the tumor cells. That is, some tissue regions are penetrated by large numbers of drug particles, whereas some tumor cells may not come in contact with sufficient concentrations of the drug particles to experience their therapeutic action. Such irregular interstitial flows also result in the faster transport of some drug particles (compare the lengths of each path shown in Figure 5B) and deeper penetration of the tissue when compared to the cases of regular cellular packing. Our simulations show that this phenomenon is more pronounced in tissues of higher cellular density. On average, across irregular tissues of the same cellular density and porosity, the permeation times are comparable to those in the regularly packed tissues (data not shown). However, for tissues in which there are multiple paths of high tortuosity, the permeation depth and time may be significantly higher.

### Permeation under a combination of advective and diffusive transports

**Figure 6:**
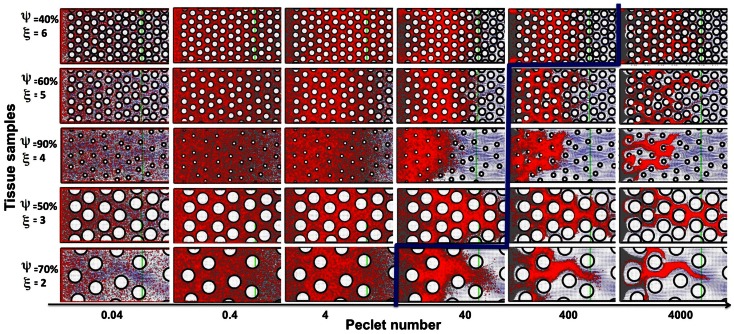
**Classification of particle traces for a combination of advective and diffusive transports**. The model parameter space separated into diffusion (left-top) or advection (right-bottom) dominated particle transport (the separation shown in blue), shown for five representative tissue samples selected out of 30 considered in this study. For the small Péclet numbers (*Pe* = 0.04–4) the transport is diffusion-dominated for all tissue topologies. For the maximal considered Péclet number (*Pe* = 4000) the transport is convection-driven for all tissue architectures. In the case of the medium Péclet numbers (*Pe* = 40–400) the particle transport changes its characteristics depending on the tissue architecture.

Drug and imaging particles, like metabolites and other molecules, are capable of random motion when suspended in a medium such as interstitial fluid. The extent and speed of this intrinsic, diffusion-type particle motility depend on the particle’s molecular mass (Einstein relation). Here we consider a wide range of particle diffusion coefficients that cover both the small molecules of metabolites (such as oxygen or glucose), large nanoparticles (designed as carriers of therapeutic compounds), and all modalities in between (Nugent and Jain, 1984; Pluen et al., 2001; Avgoustiniatos et al., 2007; Schmidt and Wittrup, 2009). We tested six different values of particle diffusion coefficients in the range of 2.5 × 10^−8^ to 10^−3^ mm^2^/s. As expected, we saw both transport phenomena: diffusion driven particle dispersal (for the Péclet numbers of 0.04–4), and advection-dominated particle relocation (for the Péclet numbers of 400–4000). The value of the Péclet number is a measure of the ratio between the advective displacement of the particles moved by the flow to the rate of the particle diffusion driven by an appropriate gradient (*Pe* = *L* × *U/D*, where *D* is a diffusion coefficient, and *L* and *U* are the characteristic values of length and velocity, respectively used to determine the Reynolds number *Re* in The Mathematical Model section). We observed that for small Péclet numbers (*Pe* = 0.04–4), the transport of particles was clearly diffusion-dominated in all considered tissues, for all values of tissue cellular density and porosity, and for both regularly and irregularly spaced cells. In all cases the entire interstitium was covered by drug particles (examples shown in Figure 6, left subspace). For the maximal Péclet number considered here (*Pe* = 4000), the transport was advection-driven, and all drug particles followed the high velocity corridors with minimal dispersion due to low diffusive properties (examples shown in Figure 6, right column). However for the values of the Péclet number of *Pe* = 40 and 400, the transport of the drug particles had different characteristics depending on the tissue structure. In the case of *Pe* = 40, only the tissues of low cellular density (ξ = 2) showed dominant advective transport. For the tissues with higher cellular densities the drug particles only followed the fluid flow initially, and their diffusive capacities became finally predominant (examples shown in Figure 6). In the case of *Pe* = 400, only the tissues with a high cellular density (ξ = 6) showed diffusive characteristics. For all other tissues the transport was dominated by the interstitial fluid flow (Figure 6). Moreover, the average tortuosity of the drug particle traces for advection-dominated transport (*Pe* = 4000, all tissue samples) was equal to τ = 1.35 which is similar to the average for the pure advection transport discussed above. In the cases of a small Péclet number, the average tortuosity is an order of magnitude larger: τ = 11.1 (with the mean value of τ = 17.9, 10.7, 4.7 for *Pe* = 0.04, 0.4, and 4, respectively).

### Comparison with permeation of targeted imaging agents

A direct comparison of our computational results with the drug distribution in tumor tissues is difficult. The *in vitro* experiments with either the 3D multicellular spheroids (Nederman et al., 1981; Walenta et al., 2002; Bryce et al., 2009) or multilayered tissue constructs (Kyle et al., 2004; Grantab et al., 2006; Modok et al., 2007; Al-Abd et al., 2008) do not reproduce the *in vivo* conditions faithfully (for example, the differences in interstitial pressure and interstitial fluid flow patterns are usually not captured). The effects of the drug actions on individual tumor cells in mouse models can be captured *ex vivo* by using the immunohistochemical staining of dead cells (Sun et al., 2012), or *in vivo* by using fluorescent drugs (Ozols et al., 1979; Lankelma et al., 1999; Primeau et al., 2005). However, intrinsically fluorescent drugs are scarce (doxorubicin, adriamycin); therefore, we will compare the results of our simulations to the penetration of fluorescent imaging agents targeted to bind to the specific cell membrane receptors expressed by some tumor cells.

**Figure 7:**
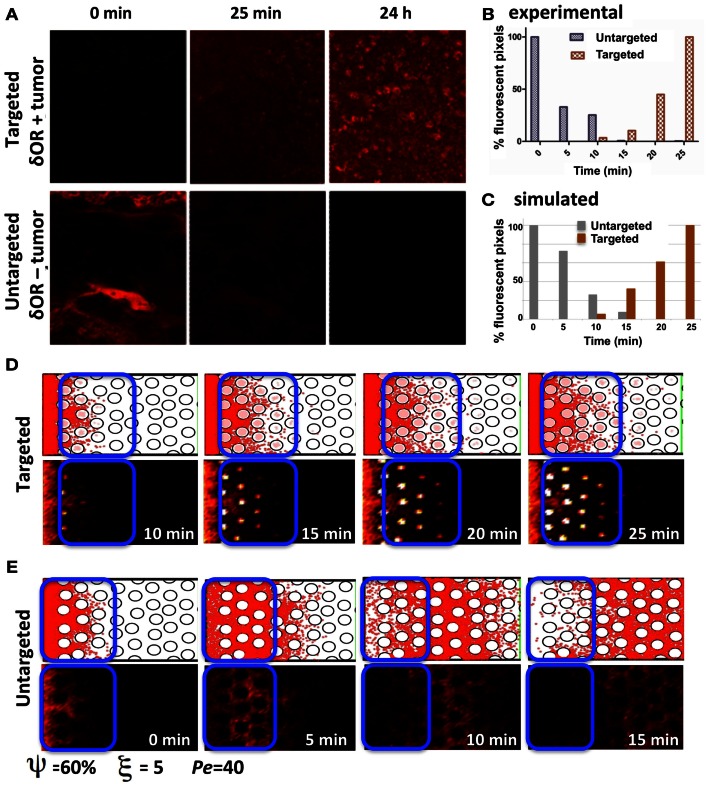
**Penetration of targeted and untargeted imaging agents: comparison of simulated and experimental data**. **(A)** Confocal fluorescence microscopy images of target-expressing DWC tumors (top) and non-expressing tumors (bottom) at time-points immediately following the injection of the targeted fluorescent probe. **(B)** For both the targeted and untargeted tumors, the bar graph represents the percentage of pixels with fluorescence intensities above a threshold value higher than the low background signal over a 25 min time-course, with 100% being equal to largest number of pixels above the background in the time-course. **(C)** The quantification of the simulated results from the section of the tissue indicated as a blue window in **(D,E)**. The simulated data has been normalized as in **(B)** for comparison. **(D)** The time sequence from a simulation with cellular uptake via receptor binding (targeted); the top images show individual particles, and the bottom images show the corresponding fluorescent rendering. **(E)** Time sequence from a simulation without cellular uptake (untargeted). Both simulations were run for the same tissue structure with a cellular density of ξ = 5, cellular porosity of ψ = 60%, and Péclet number of *Pe* = 40.

Our experimental model of choice is the DWC that is surgically implanted on a mouse dorsal-skin flap and allows for monitoring the growth of xenograft tumors over time. When combined with a targeted fluorescent imaging agent (Dmt-Tic-Cy5) we can observe agent extravasation from the vascular system, its spread through the interstitial space, cellular uptake by tumor cells that express the target marker, and agent clearance from the tissue. Two different tumors were implanted, one that expresses and a second that does not express the targeted receptor. The tumors were imaged at different time-points following the administration of the agent (Figure 7A), and the fluorescence intensities of both tumor tissue types were recorded (Figure 7B). Clearly, the tumor expressing the targeted receptor (δOR+, Figure 7A, top row) showed the slow but steady accumulation of the fluorescent agent over the period of 24 h. For the tumor that did not express the targeted receptor (δOR−, Figure 7A, bottom row) the fluorescent agent was clearly visible in the mouse veins just after injection (Figure 7A, bottom row, left image); however, since it was not absorbed by the tumor cells, it cleared from the tumor tissue in about 10 min (Figures 7A,B, bottom row middle image). For simplicity, we called the latter case “untargeted,” since the imaging agent was not able to bind to the target cell membrane receptors.

The process of imaging agent molecule uptake via binding to cell membrane receptors was incorporated into our computational model by trapping these particles that come very close to the cell boundary points inside of the cell. The course of the time for a simulation of the targeted agent with cellular uptake is shown in Figure 7D. The top images show the individual particles inside of the cells (pink dots) and in the interstitial space (red dots). The bottom images show the corresponding fluorescent rendering that was created by dividing the whole computational domain into small square grids and counting the particles inside. The obtained particle concentration is presented as a heat map. Figure 7E shows the time sequence from a simulation without cellular uptake (untargeted). Both cases were run for the same tissue structure of cellular density ξ = 5, cellular porosity ψ = 60%, and Péclet number *Pe* = 40.

In order to compare the simulated and experimental results we selected a small tissue section near the agent supply (vein) in the untargeted case and far from the vein in the targeted case. This is consistent with the way the experimental data was quantified. Again, following the experimental procedure, we counted all of the particles inside the selected reference window (indicated by blue rectangles in Figures 7D,E) and normalized the obtained counts by the maximal value from the whole time-course separately for each simulation. The quantification of the simulated results is shown in Figure 7C. Our results show a trend similar to the experimental data in the dynamics of both the targeted and untargeted agents. However, this comparison has only a qualitative value, because our computational model has not been tuned to the experimental setup, and certain model parameters (such as agent molecular binding or the level of intracellular agent saturation) have not been calibrated to match those in the experiments. Both the experimental and simulated data need to be analyzed further in order to compare the results quantitatively.

## Conclusion and Discussion

In this paper we investigated how the structure and distribution of tumor cells influence the interstitial fluid flow and the delivery of chemical compounds, such as drug or imaging particles. We used a computational model based on the method of the regularized Stokeslets of Cortez (2001) that allows for modeling both the advective transport (with the interstitial flow due to the pressure differences between the vascular system and the tumor tissue), and the diffusive transport due to the Brownian motion of the particles. While the method of regularized Stokeslets has been used previously to model various swimming organisms (Flores et al., 2005; Cisneros et al., 2007) and biofilm dynamics (Cogan et al., 2005; Cogan, 2008, 2010), to our knowledge, it has not been applied in the studies of interstitial transport through the tumor tissues.

We focused in this paper on the interplay between both drug advective and diffusive modes of transport and the structure of the tissue, taking into account both the tissue cellular density and the extent of the interstitial space between the individual cells. The advective component of the interstitial penetration is especially important for the transport of particles characterized by larger molecular weights, such as certain drug molecules or imaging nanoparticles that usually have smaller diffusion coefficients. This is in contrast to small molecules, such as oxygen or glucose, which can move through the tissue solely by the diffusive process.

The results presented in this paper show that tumor cell distribution is characterized by tissue cellular porosity and its cellular density influences the depth of a drug’s advective penetration in a non-linear manner, with sparsely packed tissues showing slower interstitial fluid flow and longer times of drug penetration when compared to more densely packed tissues. These results were obtained under the assumption that the fluid influx from the blood capillary is constant and the same in each case considered here, that is for each *in silico* tissue geometry. For simplicity, we directly imposed a specific fluid influx value on all our computational simulations. However, this parameter could be related to experimentally measurable quantities, such as the pressure differences between the capillary and the surrounding tissue or the tumor tissue. We also showed that irregularities in tissue composition and cell spatial configuration result in the emergence of tissue zones that have a lesser exposure to the drug molecules. This, in turn, may result in drug concentrations insufficient to provide therapeutic action. It has been suggested previously (Minchinton and Tannock, 2006) that the poor penetration of the tumor tissue by drug particles may leave some cell populations untreated and capable of initiating tumor regrowth or recurrence.

Our simulations also showed that tissues of higher irregular architecture were characterized by faster transport of some drug particles. Consequently, these particles were able to penetrate deeper into the tumor tissue and exert their therapeutic effects on a larger tissue area. Thus, we observed a certain dichotomy in our simulations. The advection-dominated drug particle transport in tissues of highly irregular architectures resulted in tissue regions of permanent low drug exposure even near the vascular system, and tissue regions far from the vascular system that were exposed to the drug action temporally. Thus drugs of higher diffusivity (smaller molecular size) were able to penetrate the tissue more uniformly, potentially bringing an effective treatment to all cells, but only near the vasculature. On the other hand, drugs of lower diffusivity were able to reach distant parts of the tissue. As a result, they could have an extended beneficial effect, but only if they can be absorbed quickly by the cells, or have a longer half-life time in order to allow for the drug accumulation to exert its lethal effects. These kinds of physico-chemical properties of individual molecules may be beneficial for the imaging agents that are not meant to kill the cells, thus small concentrations accumulated inside the cells may still fluorescently mark the tumor cells located farther from the vasculature. However, in this case, the biomarkers need to bind to the specific membrane receptors quickly and have longer half-life times.

The research studies initiated in this paper are novel in the areas of computational drug design and bio-medical modeling. We proposed to investigate an important but overlooked area in testing anticancer drug efficacy: the effective penetration of tumor tissues by drug particles under various extrinsic conditions. Most research approaches addressing drug efficacy have concentrated on the molecular and genetic mechanisms of chemical compounds, whereas the role of the tumor microenvironment as a limiting factor in drug distribution has received much less attention. Moreover, the majority of *in silico* methods for assessing the ADME-T properties (absorption, distribution, metabolism, excretion, and toxicity) of pharmaceutical compounds do not consider the spatial aspects of drug pharmacokinetics and pharmacodynamics (PK/PD), but instead, treat the blood and all organs as well-mixed compartments neglecting their natural heterogeneity. It is interesting to note that the question of how the architecture of natural or fabricated obstacles influences the interstitial transport has been addressed in other scientific areas, including groundwater hydrology (Kang et al., 2010; Xie et al., 2011; Tsimpanogiannis and Lichtner, 2012), vortex dynamics in superconductors (Nori, 1996; Reinchardt et al., 1997; Silhanek et al., 2010), fuel conversion (Machado, 2012), and biofilm dynamics (Dillon and Fauci, 2000).

**Figure 8:**
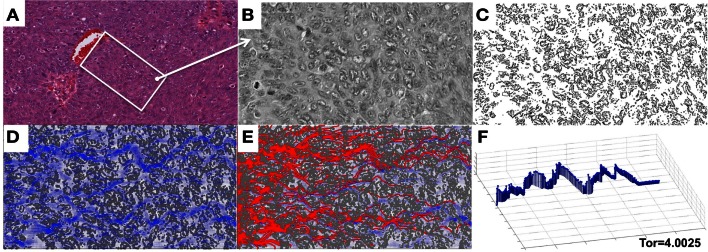
**Penetration of drug particles through a digitized ovarian tumor tissue**. **(A)** An original H&E-stained histological image of ovarian tumor tissue with a centrally located vein. **(B)** A small patch of tissue selected from **(A)** and used for digitization. **(C)** A digitized version of the histological image from **(B)** used for computational simulations. **(D)** The simulated interstitial fluid flow (blue). **(E)** The resulting traces of the simulated particles representing the drug or biomarker molecules. **(F)** The particle trace with maximal tortuosity of 4.0025.

We focused in this study on analyzing the permeation properties of tissues characterized by different cellular architectures, but with identical circular cells. However, as can be seen in Figure 1, the realistic tumor tissues are far more complex. They are composed of cells that vary not only in their spatial configuration, but also in their size, shape, and receptor expression. In such highly irregular tissues the patterns of interstitial transport can be even more convoluted. To illustrate this, we applied our model to a digitized histological sample from human breast cancer tissue. Figure 8 shows the original histological image of a small patch of tumor tissue, a digitized version of this image used for computational simulations, and the resulting fluid field and traces of the drug/biomarker particles. The maximal tortuosity is reported.

We also presented a qualitative comparison of the results from our model simulations with the data from the DWC experiments. However, in order to achieve the quantitative results, the model needs to be parameterized to reproduce the properties of a given tumor and the properties of a given drug or (un)targeted imaging agent. Fluorescently labeled probes, together with the DWC, form an ideal model for taking *in vivo* measurements at various time-points in the same animal. While certain measurements of these probes can be obtained *in vitro* (such as association and dissociation binding constants, mass and size, saturation levels, and cellular uptake), the fluorescent microscopy of DWC tumor xenografts enables observation of probe interactions within a living tumor microenvironment. Mathematical modeling and analysis of these fluorescence image acquisitions allows for the estimation of the relative probe concentration in plasma and tumor tissue, rate of probe extravasation and penetration into the tumor, rate of cellular binding and internalization, and rate of vessel clearance. The tissue architecture can be determined from the *ex vivo* histological images as they are shown in Figure 8, and then used to fit the model parameters, i.e., interstitial velocity and probe effective diffusion. This can be done by performing multiple simulations with systematically varied parameters and then comparing the simulated results to intravital or *ex vivo* images.

The model presented here constitutes the basis for further extensions that will increase the model’s realism by including several factors specific to either the drug/biomarker or the microenvironment. We plan to explicitly model drug particle size, mass, and electrical charge. Our oversimplified model of cellular uptake will be extended to incorporate the mechanisms of cellular efflux and influx, as well as the more elaborate models of molecular binding, including receptor-ligand reaction kinetics. Our future model extensions will also include models of different structures of the ECM, such as the extracellular fiber distribution and alignment. One of the important aspects of modeling anticancer drug actions is to integrate both cellular death and growth into the model, and to simulate much longer time regimes in order to test the tumor eradication or recurrence. The model could also be extended into the full three-dimensional space [the appropriate blob functions have been proposed previously (Cortez, 2001)]. The full version of this model will provide a tool for testing drug efficacy by independently varying the drug and tissue parameters over a wide range of values that are often difficult to replicate in laboratory experiments.

## Conflict of Interest Statement

The authors declare that the research was conducted in the absence of any commercial or financial relationships that could be construed as a potential conflict of interest.
